# Roles of dopamine neurons in mediating the prediction error in aversive learning in insects

**DOI:** 10.1038/s41598-017-14473-y

**Published:** 2017-10-31

**Authors:** Kanta Terao, Makoto Mizunami

**Affiliations:** 1 Graduate School of Life Science, Hokkaido University, Sapporo, 060-0810 Japan; 2 Faculty of Science, Hokkaido University, Sapporo, 060-0810 Japan

## Abstract

In associative learning in mammals, it is widely accepted that the discrepancy, or error, between actual and predicted reward determines whether learning occurs. The prediction error theory has been proposed to account for the finding of a blocking phenomenon, in which pairing of a stimulus X with an unconditioned stimulus (US) could block subsequent association of a second stimulus Y to the US when the two stimuli were paired in compound with the same US. Evidence for this theory, however, has been imperfect since blocking can also be accounted for by competitive theories. We recently reported blocking in classical conditioning of an odor with water reward in crickets. We also reported an “auto-blocking” phenomenon in appetitive learning, which supported the prediction error theory and rejected alternative theories. The presence of auto-blocking also suggested that octopamine neurons mediate reward prediction error signals. Here we show that blocking and auto-blocking occur in aversive learning to associate an odor with salt water (US) in crickets, and our results suggest that dopamine neurons mediate aversive prediction error signals. We conclude that the prediction error theory is applicable to both appetitive learning and aversive learning in insects.

## Introduction

Associative learning allows animals to adapt to various environments by acquiring knowledge on events in their environments. Based on the knowledge, animals find suitable food, avoid toxic food and escape from predators. Thus, both appetitive learning and aversive learning are essential for survival of animals. Many efforts have been made to elucidate learning rules governing associative learning in mammals^1,2^, but whether appetitive learning and aversive learning are ruled by the same general principles remains unclear.

In associative learning in mammals, it is widely accepted that the discrepancy, or error, between the actual unconditioned stimulus (US) and predicted US determines whether learning occurs when a stimulus is paired with the US^1,2^. This theory stems from the finding of a “blocking” phenomenon by Kamin^3^. He observed in rats that a stimulus X that had been paired previously with a US could block subsequent association of a second stimulus Y to the US when the two stimuli were paired in compound with the same US (XY + training, see Table 1). Kamin^3^ argued that no learning of stimulus Y occurs since the US was fully predicted by stimulus X and argued that surprise is needed for learning. This proposition was formulated into the prediction error theory by Rescorla and Wagner^4^, and subsequent electrophysiological studies suggested that dopamine (DA) neurons in the midbrain convey reward prediction error signals^1^.

**Table 1:** Procedures used for and results of compound conditioning, blocking and auto-blocking experiments.

Group	Phase 1	Phase 2	Results: Learning of Y?	Figures
Compound	—	XY+	Yes	Fig. 2
Control	—	Y+	Yes	Fig. 2
Blocking	X+	XY+	No	Fig. 3
Unpaired control	X/+	XY+	Yes	Fig. 3
Auto-blocking	Y + (under flupentixol)	Y+	No	Fig. 5
Control	Y/ + (under flupentixol)	Y+	Yes	Fig. 5
Auto-blocking	Y + (under epinastine)	Y+	Yes	Fig. S1

XY+: a compound of stimulus X and stimulus Y is paired with aversive US; Y/+ : unpaired presentation of stimulus Y and aversive US.

Evidence for the prediction error theory, however, has been imperfect since blocking can also be accounted for by theories other than the prediction error theory such as attentional theory and retrieval theory^5–7^, which account for blocking by competition between X and Y stimuli, and evidence to convincingly refute alternative theories has been lacking^8–10^.

We previously reported blocking in appetitive associative learning in crickets^11^. Moreover, we obtained evidence that octopamine (OA) neurons play critical roles in appetitive learning in crickets^12–19^, and we demonstrated that when a stimulus X was paired with water (appetitive US) under the condition of administration of an OA receptor antagonist, in which no learning of X occurs, subsequent learning of X was blocked in training to associate the stimulus X with the US given after recovery from the effect of the antagonist^11^. This “auto-blocking” can be accounted for by the prediction error theory since if blockade of OA-ergic transmission impairs learning but not formation of the prediction of the US by stimulus X, no learning of stimulus X should occur in subsequent training. This “auto-blocking” phenomenon cannot be accounted for by any of the competitive theories to account for blocking since it occurs without stimulus competition. Therefore, demonstration of blocking and auto-blocking phenomena in the same learning paradigm in the same species provided rigorous evidence for the prediction error theory in appetitive learning. In addition, the results of an auto-blocking experiment suggested that OA neurons mediate reward prediction error signals in crickets. However, rigorous evidence to show the applicability of the prediction error theory in aversive learning has been still lacking.

In the present study, we investigated whether blocking and auto-blocking occur in aversive learning in crickets. We have shown that DA neurons play critical roles in aversive learning in crickets^12–19^, as has been reported for other invertebrates^20–23^. We obtained evidence of blocking in conditioning to associate an odor or pattern with NaCl solution (aversive US) in crickets. We also found that “auto-blocking” occurs in aversive learning, that is, no learning of an odor X occurs in training to associate X with aversive US when the training is preceded by the same training under the condition of administration of a DA receptor antagonist. This blockade of learning was accounted for by the prediction error theory but not by alternative theories to account for blocking since no cue competition is involved.

## Results

### Effects of compound conditioning

**Figure 1:**
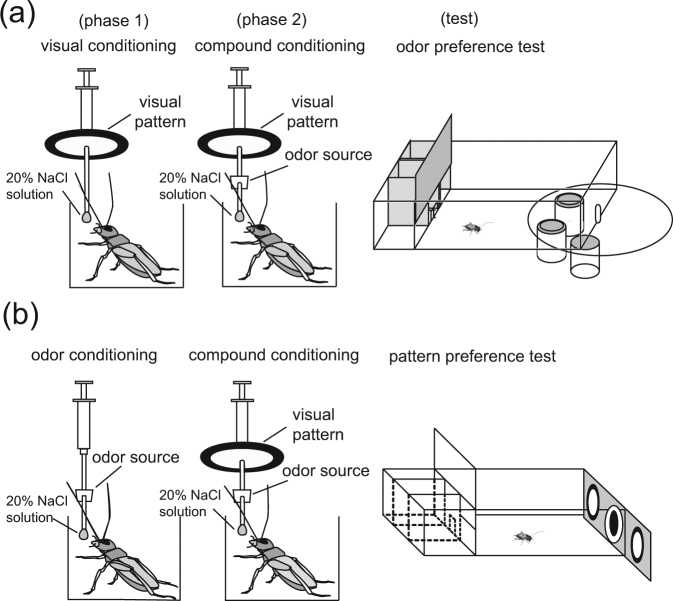
Experimental procedures for blocking of olfactory learning (**a**) or visual learning **(b)**. (**a**) A group of water-deprived crickets, individually placed in a beaker, was subjected to pairings of a visual pattern with NaCl solution (P+ training) and then pairings of an odor-pattern compound with NaCl solution (OP+ training). Relative preference for the conditioned odor compared with a control odor was tested before and after training in a test chamber. (**b**) Another group of crickets was subjected to pairings of an odor with NaCl solution (O+ training) and then pairings of an odor-pattern compound with NaCl solution (OP+ training). Relative preference for the conditioned pattern compared with a control pattern was tested before and after training in a test chamber. The figures were modified from our previous paper^11^. https://creativecommons.org/licenses/by/4.0/.

**Figure 2:**
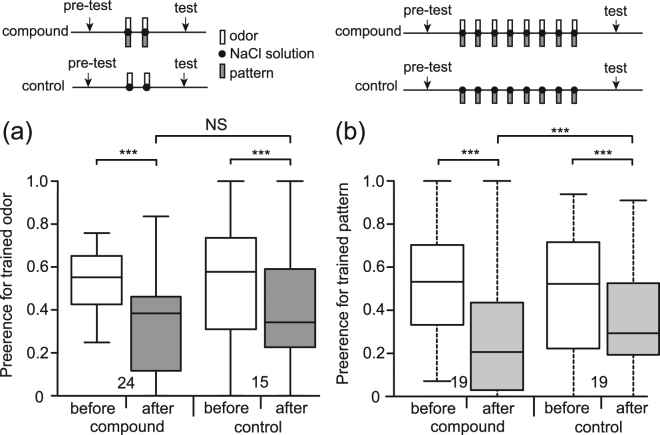
Effect of odor-pattern compound conditioning. (**a**) One group of animals (compound group) was subjected to 2-trial pairing of an odor-pattern compound with NaCl solution (aversive US) and another group (control group) was subjected to 2-trial pairing of an odor alone with NaCl solution. (**b**) One group of animals (compound group) was subjected to 8-trial pairing of an odor-pattern compound with NaCl solution and another group (control group) was subjected to 8-trial pairing of a pattern alone with NaCl solution. The inter-trial interval (ITI) was 5 min. Relative preference for the odor or pattern was tested before and at 20 min after training. The experimental procedures are illustrated at the top, and relative preferences for the trained odor or pattern before training (white boxes) and after training (grey boxes) are shown as box and whisker diagrams. The horizontal line in the box is the median, and the box represents the 25-75 percentiles in this and in all following figures. Whiskers extend to extreme values as long as they are within a range of 1.5 × box length. The outliers are shown as open circles. The number of animals is shown below the boxes. A GLMM was used to examine relative preferences for the trained odor or pattern before and after training in the compound and control groups (Supplemental Table S1). Statistical significance is shown as asterisks (***p < 0.001; NS p > 0.05).

Since a blocking experiment requires conditioning of two stimuli presented at the same time, we first investigated whether crickets exhibit such compound conditioning. We used odor-pattern compound conditioning (OP+ conditioning), in which a compound stimulus consisting of an odor (O) and a visual pattern (P) is paired with a 20% NaCl solution (aversive US) (+), and we investigated whether OP+ training leads to learning of the odor or the visual pattern (Fig. 1 and Table 1). One group of animals (compound group) was subjected to 2-trial OP+ training and another group (control group) was subjected to 2-trial olfactory conditioning (O+ conditioning). Relative preference for the odor used in training compared to the control odor was tested before and at 20 min after training in both groups. The results are shown in Fig. 2a. We used a generalized linear mixed model (GLMM) to evaluate the data (see Methods). Both the compound group and control group exhibited significantly decreased preference for the conditioned odor after training compared to that before training (test term, p = 8.68 * 10^−10^, z = −6.132, see Supplemental Table S1). The preference for the conditioned odor after training in the compound group did not significantly differ from that in the control group (test * training term, p = 0.141, z = 1.471). The results showed that learning was achieved in the compound group as in the control group. We thus conclude that odor-pattern compound conditioning leads to conditioning of the odor.

Next, we investigated whether OP+ training leads to learning of the visual pattern. One group of animals (compound group) was subjected to 8-trial OP+ training and another group (control group) was subjected to 8-trial visual conditioning (P+ conditioning). Relative preference for the pattern used in training compared to the control pattern was tested before and at 20 min after training in both groups. The results are shown in Fig. 2b. Both the compound group and control group exhibited significantly decreased preference for the conditioned pattern after training compared to that before training (test term, p = 7.97 * 10^−15^, z = −7.768). The results showed that learning was achieved in both the compound group and the control group. In addition, we observed that the preference for the conditioned pattern after training in the compound group was significantly less than that in the control group (test * training term, p = 1.35 * 10^−4^, z = 3.818). This was unexpected since we did not observe such effect in appetitive visual learning as is discussed in a later section.

### Demonstration of blocking

**Figure 3:**
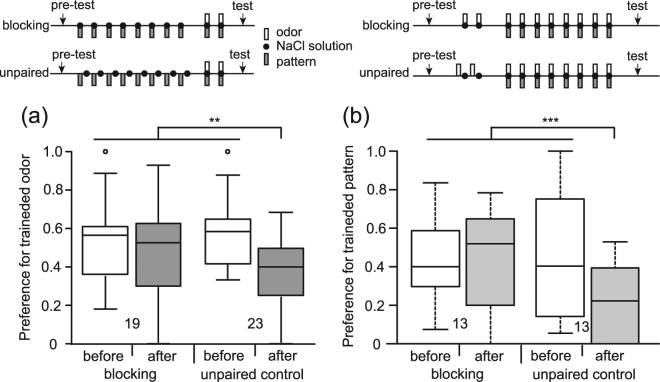
Blocking of olfactory learning (**a**) or visual learning **(b)**. (**a**) One group of animals (blocking group) was subjected to 8-trial pairing of a pattern with NaCl solution, and 20 min later the group was subjected to 2-trial pairing of an odor-pattern compound with NaCl solution. Another group (unpaired control group) was subjected to unpaired presentation of a pattern and NaCl solution 8 times each, and 20 min later the group was subjected to 2-trial pairing of an odor-pattern compound with NaCl solution. (**b**) One group of animals (blocking group) was subjected to 2-trial pairing of an odor with NaCl solution, and 20 min later the group was subjected to 8-trial pairing of an odor-pattern compound with NaCl solution. Another group (unpaired control group) was subjected to unpaired presentation of an odor and NaCl solution 2 times each, and 20 min later the group was subjected to 8-trial pairing of an odor-pattern compound with NaCl solution. The ITI was 5 min. Relative odor or pattern preference was tested before and at 20 min after training. Relative preferences for the trained odor or pattern before (white bars) and after (gray boxes) training are shown as box and whisker diagrams. The number of animals is shown below the boxes. A GLMM was used to examine relative preferences for the conditioned odor or pattern before and after conditioning in the blocking and unpaired control groups (Supplemental Table S1). Statistical significance is shown as asterisks (**p < 0.01; ***p < 0.001).

We next studied whether blocking occurs in aversive learning in crickets. At first, we investigated whether blocking of olfactory learning occurs. One group of crickets (blocking group) was subjected to 8-trial P+ training and then 2-trial OP+ training (Table 1). Another group (control group) was subjected to unpaired presentations of a visual pattern and aversive US (P/+ training) 8 times each and then 2-trial OP+ training. The results are shown in Fig. 3a. The preference for the trained odor after training in the control group was significantly less than that before training in the same group and was also significantly less than that before or after training in the blocking group (test * training term, p = 0.00148, z = −3.179). The results showed that conditioning was achieved in the control group but not in the blocking group.

We next studied whether blocking of visual pattern learning occurs. One group of crickets (blocking group) was subjected to 2-trial O+ training and then 8-trial OP + training. Another group (control group) was subjected to unpaired presentations of an odor and aversive US (O/+ training) 2 times each and then 8-trial OP+ training. The results are shown in Fig. 3b. The preference for the trained pattern after training in the control group was significantly less than that before training in the same group and was also significantly less than that before or after training in the blocking group (test * training term, p = 3.6 * 10^−8^, z = −5.509). The results showed that conditioning was achieved in the control group but not in the blocking group. The results indicate that blocking occurs in visual learning.

### A neural circuit model of classical conditioning that matches the prediction error theory

**Figure 4:**
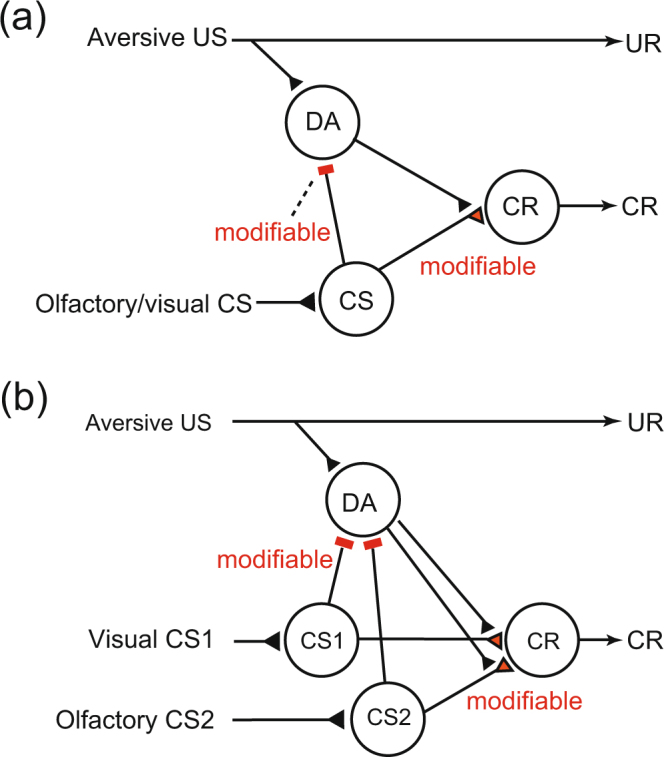
A model of aversive conditioning in crickets. (**a**) A model for the roles of DA neurons in aversive conditioning to match the prediction error theory. In the model, “DA” neurons (assuming DA neurons projecting to the mushroom body (MB) lobe) govern enhancement of synaptic transmission that underlies learning. The “DA” neurons are assumed to receive silent or very weak inhibitory synapses from “CS” neurons (assuming Kenyon cells of the MB) before training, which are strengthened by CS-US pairing. During training, “DA” neurons receive excitatory synaptic input representing actual US and inhibitory synaptic input from “CS” neurons representing US predicted by CS, and thus their activities represent US prediction errors (see Supplemental Table S2), thereby allowing US prediction error signals to govern enhancement of synaptic transmission from “CS” neurons to “CR neurons (the latter assuming output neurons from the MB lobe). A complete version of our model is described in Supplemental Figure S2. (**b**) Accounting for blocking by the model. After a sufficient number of CS1-US pairings, “DA” neurons are inhibited by activation of “CS1” neurons during pairing of a compound of CS1 and CS2 with US, and thus activities of “DA” neurons in response to US presentation are diminished. As a result, no enhancement of synapses from “CS2” neurons to “CR” neurons occurs by subsequent compound conditioning of CS1 and CS2 with the US, and thus no learning of CS2 occurs. Synapses for which efficacy can be changed by conditioning are colored in red and marked as “modifiable”. Excitatory synapses are marked as triangles; inhibitory synapses are marked as bars. UR: unconditioned response.

We previously proposed a neural circuit model for appetitive learning that matches the prediction error theory^11^. The model was designed to represent neural circuits in lobes of the MB, which is known to play critical roles in learning^20,21^, and was based on our findings that OA neurons play critical roles in appetitive learning in crickets^12–17,19^. Here we propose a model of aversive learning that matches the prediction error theory (Fig. 4), in which we focused on the roles of DA neurons in aversive learning^12–19^. For complete description of our model, see Supplementary Figure S2.

In the model shown in Fig. 4a, “DA” neurons (assuming DA neurons projecting to the lobes of the MB) are assumed to receive inhibitory synapses from “CS” neurons (assuming Kenyon cells of the MB), the efficacy of which is strengthened by conditioning. In pairing of an olfactory CS with a sodium chloride US, “DA” neurons receive excitatory input representing actual US and inhibitory input representing predicted US by the CS, and their responses thus represent US prediction error signals. Hence, US prediction error signals govern enhancement of synaptic transmission that underlies conditioning. How the model accounts for blocking is shown in Fig. 4b (for an explanation, see legends). To better account for the model, information coded by “DA” neurons before and after training is shown in Supplemental Table S2.

### Demonstration of auto-blocking

Our model predicts that blockade of synaptic transmission from DA neurons by a DA receptor antagonist (flupentixol^24^) during Y+ training impairs learning of Y but not formation of aversive US prediction by Y since, assuming that the antagonist impairs enhancement of “CS-CR” synapses but not that of “CS-DA” synapses (see Fig. 4a), subsequent Y+ training given after recovery from the effect of the antagonist should produce no learning. This effect is termed “auto-blocking”, because learning of Y is blocked by US prediction by Y itself, not by X in the case of blocking. We previously reported such an auto-blocking phenomenon in appetitive learning in crickets by using OA receptor antagonist (epinastine)^11^.

**Figure 5:**
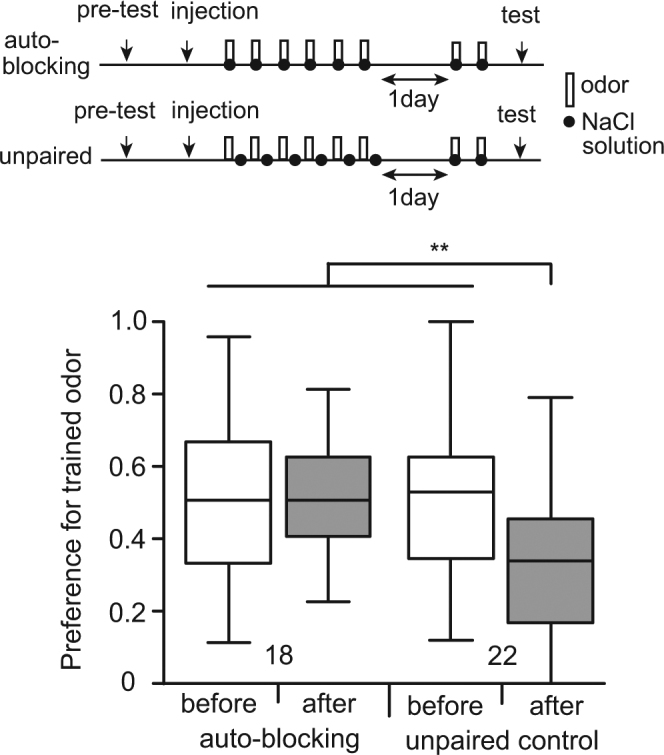
Auto-blocking. Two groups of animals received a pre-test and were then injected with 3 μl of saline containing 100 μM flupentixol. Thirty min later, one group (auto-blocking group) was subjected to 6-trial pairing of an odor with NaCl solution and the other group (unpaired control group) was subjected to unpaired presentation of an odor and NaCl solution 6 times each. The ITI was 5 min for the former and it was 2.5 min for the latter. On the next day, both groups were subjected to 2-trial pairing of the odor with NaCl solution and 20 min later they received a post-test. Relative odor preferences for the trained odor before (while boxes) and after (gray boxes) training are shown as box and whisker diagrams. The number of animals is shown below the boxes. A GLMM was used for comparison of relative preferences for the trained odor before and after conditioning in the auto-blocking and control groups (Supplemental Table S1). Statistical significance is shown as asterisks (**p < 0.01).

We tested whether auto-blocking occurs in aversive learning in crickets. One group of animals (auto-blocking group) was injected with flupentixol into the head hemolymph and 30 min later the group was subjected to 6-trial O+ training. The dose of flupentixol was determined based of our previous studies^15–17^. On the next day, the group was subjected to 2-trial O+ training. Another group (control group) was subjected to unpaired presentation of the odor and aversive US (O/+ training) 6 times each under the condition of application of flupentixol and then was subjected to 2-trial O+ training the next day. The results are shown in Fig. 5. The preference for the trained odor after training in the control group was significantly less than that before training in the same group and was significantly less than that before or after training in the auto-blocking group (test * training term, p = 0.00144, z = −3.186). The results showed that learning was achieved in the control group but not in the auto-blocking group and indicate that auto-blocking occurs in aversive learning in crickets.

We previously showed that octopamine receptor antagonist (epinastine) does not impair aversive learning^13–17^, and here we performed an experiment to confirm that epinastine does not lead to auto-blocking in aversive learning. One group of animals was injected with epinastine into the head hemolymph and 30 min later the group was subjected to 6-trial O+ training. On the next day, the group was subjected to 2-trial O+ training. The results are shown in Supplemental Figure S1. The preference for the trained odor after training was significantly less than that before training (test term, p = 7.23 * 10^−4^, z = −3.381), indicating that learning was successful. We conclude that DA receptor antagonist but not OA receptor antagonist leads to auto-blocking of aversive learning.

## Discussion

We obtained convincing evidence for the prediction error theory in aversive learning. We demonstrated, at first, that a blocking phenomenon occurs in aversive learning in crickets, i.e., no learning of Y occurred by XY+ training when the training was preceded by X+ training with X and Y being either visual or olfactory stimulus. Then we proposed a neural circuitry model of aversive learning, in which our previous model of aversive learning^15^ was modified to match the prediction error theory. Our aversive learning model (Fig. 4) was a counterpart of the appetitive learning model we proposed previously^11^ and predicted an “auto-blocking” phenomenon, in which no learning of X occurs by X+ training when the training is preceded by X+ training under the condition of administration of a DA receptor antagonist, and we indeed observed this phenomenon in olfactory learning. The results of the auto-blocking experiment showed the validity of the prediction error theory: To our knowledge, all theories to account for blocking other than the prediction error theory, including attentional theories^5,6^ and retrieval theories^7^ (or comparator hypothesis), assume cue competition between X and Y to account for blocking, and these theories thus fail to account for auto-blocking. Demonstration of blocking and auto-blocking phenomena in aversive learning (this study) and in appetitive learning^11^ in the same species provides rigorous evidence for the prediction error theory in both appetitive and aversive forms of olfactory learning in crickets. Demonstration of auto-blocking of visual learning remains for our future subject.

### Previous reports on blocking in aversive learning in animals

Blocking has been reported in various systems of aversive learning in vertebrates and invertebrates. A blocking phenomenon was first demonstrated in classical conditioning of tone and light compound stimuli with electric shock US in rats^3^. Evaluation of this learning paradigm led to proposals of the prediction error theory^4^, attentional theory^5,6^ and retrieval theory^7^. Blocking in aversive learning has also been reported in mollusks, in which odor, light or tactile stimulus was paired with bitter taste, electric shock or other aversive US^25–27^. Some researchers attempted to discriminate the prediction error theory with alternative theories to account for blocking in aversive conditioning, but convincing evidence to discriminate among different theories has not been reported^8–10^. The auto-blocking experiment described here may help to discriminate different learning theories in these animals.

We observed that the effect of compound conditioning of a visual pattern and an odor was significantly more than that of conditioning of a visual pattern (Fig. 2b), indicating that simultaneous presentation of an olfactory cue facilitated conditioning of a visual cue. This is an unexpected observation since we did not find such effect in appetitive visual conditioning^11^. Whether this effect is specific to aversive visual learning remains to be clarified.

### Roles of dopamine neurons in mediating aversive prediction error signals

DA neurons are thought to convey reinforcement signals in many systems of associative learning in insects and mammals. In the fruit-fly *Drosophila*, it has been suggested that different classes of DA neurons projecting to the lobes of the MB mediate reinforcement signals in aversive learning and appetitive learning^20,21^. In honey bees, as in crickets, it has been suggested that DA neurons convey reinforcement signals in aversive learning^22^, whereas OA neurons convey reinforcement signals in appetitive learning^28,29^. However, the exact nature of signals that DA or OA neurons convey in learning has not been characterized in any insects. Future electrophysiological studies on activities of DA neurons during conditioning are needed to clarify this issue.

In mammals, there is evidence that midbrain DA neurons mediate prediction error signals in appetitive learning^1,2,30,31^, but the roles of DA neurons in aversive learning remain controversial. Some researchers have suggested that midbrain DA neurons participate in aversive learning^32,33^ and convey aversive prediction error^34^, but other researchers have argued that midbrain neurons mediating aversive signals may not be DAergic^31,35,36^. To what extent the roles of DA neurons in associative learning are conserved between insects and mammals remains for a fascinating research subject.

### Are there interactions between neurons mediating prediction errors about reward and aversiveness?

We suggest that OA and DA neurons convey prediction error signals in appetitive learning and aversive learning, respectively, in crickets and an important future subject is to investigate whether OA and DA neurons independently process reward and aversive prediction error signals, respectively, or whether these neurons tightly interact to integrate reward and aversive prediction error signals and to form a unified system to mediate value prediction error signals in insects. We previously observed that intervention of DA-ergic transmission by DA receptor antagonists or by knockdown or knockout of genes that code for a type of DA receptor by RNAi or by the CRISPR/cas9 system impaired aversive learning but did not affect appetitive learning, whereas intervention of OA-ergic transmission impaired appetitive learning but not aversive learning^12–19^. In this study, we showed that DA receptor antagonist but not OA receptor antagonist leads to auto-blocking of aversive learning. The results indicate that the OA reward system and DA aversion system can act independently when appetitive learning and aversive learning occur independently. Those studies, however, do not exclude the possibility that DA and OA neurons interact in a situation in which a stimulus is associated with appetitive and aversive stimuli. A similar issue has been discussed in mammals. Some researchers have suggested that separate classes of midbrain neurons mediate prediction error signals about reward and  aversiveness ^31,35,36^, whereas other researchers have proposed that a single class of DA neurons integrates reward and aversive signals to encode value prediction error signals^34^. Further investigations in insects may help to better clarify this issue.

We conclude that insects predict future biologically significant events by appetitive and aversive associative learning and that DA neurons mediate prediction error signals in aversive learning. Neural circuitry mechanisms for computation of the prediction error remain unknown in any animals, and insects should emerge as pertinent models in which to elucidate this important subject.

## Methods

### Insects

Adult male crickets, *Gryllus bimaculatus*, at 1 week after the imaginal molt were used. Before the experiment, animals were placed individually in beakers and deprived of drinking water for 4 days to enhance their motivation to search for water.

### Olfactory and Visual Conditioning Procedures

We used classical conditioning and operant testing procedures described previously^11,37^ (Fig. 1). In olfactory conditioning, maple or vanilla odor (conditioned stimulus, CS) was paired with NaCl solution (aversive US). In visual conditioning, a white-center and black-surround pattern (white-center pattern) was paired with 20% NaCl solution. The outer diameter of the pattern was 4 cm and that of the while center pattern was 3 cm. In the compound conditioning, an odor and a white-center pattern were presented simultaneously (compound CSs) and were paired with NaCl solution. A syringe was used to present the CS and US to each cricket. The syringe contained NaCl solution as US, and at its needle, a filter paper soaked with odor essence was attached as olfactory CS, and/or a white-center pattern was attached as visual CS (Fig. 1). For a conditioning trial, an odor was approached to the antennae (within 1–2 cm) or a visual pattern was approached to the head of the cricket (within 2–3 cm) and held for 3 sec, and then a drop of NaCl solution was attached to its mouth. For an unpaired trial, an odor or a visual pattern was approached to the antennae or the head and held for 3 sec, and 2.5 min later, a drop of NaCl solution was attached to its mouth by another syringe. In all pairing experiments, the intervals between the trials (inter-trial intervals, ITIs) were 5 min. After olfactory or compound conditioning trials, the air in the beaker was ventilated.

### Preference Tests

Odor preference tests were carried out as described previously^11,37^. All groups were tested with relative preference between the maple odor and vanilla odor before conditioning and 20 min or 1 day after conditioning. The test apparatus consisted of waiting chambers and a test chamber. The floor of the test chamber had two holes that connected the chamber with two cylindrical containers that contained a filter paper soaked with either maple or vanilla essence and was covered with a fine gauze net (Fig. 1). Three containers were mounted on a rotative holder, and two of the three containers could be located simultaneously beneath the holes of the test chamber. Before testing, a cricket was transferred to the waiting chamber and left for about 4 min to become accustomed to the surroundings. Then the cricket was allowed to enter the test chamber and the test started. Two min after the test had started, the relative positions of the odor sources were changed by rotating the container holder. The preference test lasted for 4 min. We considered that the cricket visited an odor source when the cricket probed the top net with its mouth or palpi. The time that the cricket visited each odor sources was recorded cumulatively for each seconds. If the total visiting time of a cricket to odor sources was less than 10 sec, we considered that the animal was less motivated, possibly due to a poor physical condition, and the data were rejected. In the present experiments, about 15% animals were rejected in each test.

### Pharmacology

Crickets were injected with 3 μl of saline containing 100 μM flupentixol or 2 μM epinastine (Sigma-Aldrich, Tokyo) into the head hemolymph 30 min before the training. The estimated final concentration after circulation is 350 nM for flupentixol and 7.0 nM for epinastine^11,12^.

### Statistical Analysis

Relative preference for the conditioned odor compared with the control odor was determined as the proportion of time spent visiting the conditioned odor in the total time spent visiting the two odors. We measured the search time with the accuracy of seconds. In our previous study, we used non-parametric statistical tests for evaluation of the relative preference. Since it has been proposed that the use of a generalized linear mixed model (GLMM) is advantageous for evaluation of biological data^38^, we used a GLMM with a binomial distribution of the relative preference, determined by the search time data sampled for each second, and logit link function. We included the test condition (test before or after training), training procedure and the interaction term (test * training) as fixed effects in the GLMM, with the training and test terms being categorical variables. Individual cricket was used as a random effect, allowing the random intercept. We used R (ver. 3. 3. 1) and lme4 (ver. 1.1.12) packages for statistical analysis. We refer to as “significantly different” if p-values in the Wald statistical test in the GLMM were p < 0.05.

## Electronic supplementary material

Supplementary materials
