# Supplementary material for: Roles of dopamine neurons in mediating the prediction error in aversive learning in insects
Source: Sci Rep. 2017 Oct 31;7:14694. doi: 10.1038/s41598-017-14473-y (PMC5665953; doi:10.1038/s41598-017-14473-y)
Supplement: Supplementary file 1 — Supplementary materials [file 41598_2017_14473_MOESM1_ESM.pdf]

Title: Roles of dopamine neurons in mediating the prediction error in aversive learning in insects.

Authors: Kanta Terao and Makoto Mizunami

Supplementary Table S1 and S2
Supplementary Table S2
Supplementary Figure S1
Supplementary Figure S2

Supplemental table S1. Summary of generalized linear mixed model (GLMM).

a. Compound conditioning experiment for olfactory learning (Fig. 2a)

Fixed effects	Estimate	Standard error	Z value	P value
Intercept	0.2939	0.1913	1.537	0.124
Test	-0.9557	0.1559	-6.132	$8.68 * 10^{-10}$
Training	-0.08869	0.2446	-0.363	0.717
Test * Training	0.2947	0.2004	1.471	0.141

b. Compound conditioning experiment for visual learning (Fig. 2b)

Fixed effects	Estimate	Standard error	Z value	P value
Intercept	0.1952	0.2809	0.695	0.487
Test	-1.2552	0.1616	-7.768	$7.97 *10^{-15}$
Training	-0.4594	0.3954	-1.162	0.245
Test * Training	0.7840	0.2053	3.818	$1.35 *10^{-4}$

c. Blocking experiment for olfactory learning (Fig. 3a)

Fixed effects	Estimate	Standard error	Z value	P value
Intercept	0.1639	0.1398	1.173	0.241
Test	-0.1943	0.1455	-1.336	0.182
Training	0.06987	0.1930	0.362	0.717
Test * Training	-0.6110	0.1922	-3.179	0.00148

d. Blocking experiment for visual learning (Fig. 3b)

Fixed effects	Estimate	Standard error	Z value	P value
Intercept	-0.2931	0.2954	-0.992	0.321
Test	0.1979	0.1573	1.258	0.208
Training	-0.1123	0.4255	-0.264	0.792
Test * Training	-1.330	0.2414	-5.509	$3.6 * 10^{-8}$

e. Auto-blocking experiment with flupentixol in olfactory learning (Fig. 5)

Fixed effects	Estimate	Standard error	Z value	P value
Intercept	0.03878	0.1618	0.240	0.811

Test	-0.01699	0.1348	-0.126	0.900
Training	-0.007372	0.2152	-0.034	0.973
Test * Training	0.5831	0.1830	-3.186	0.00144

f. Auto-blocking experiment with epinastine in olfactory learning (Supplemental Fig. S1)

Fixed effects	Estimate	Standard error	Z value	P value
Intercept	0.2268	0.2314	0.980	0.327
Training	-0.5928	0.1753	-3.381	$7.23 * 10^{-4}$

By using a GLMM, effects of the test situation (before or after training), training procedure (compound conditioning or control procedure in a and b, blocking or control procedure in c and d, auto-blocking or control procedure in e) and interaction between the test and training on relative preference for the trained odor or pattern were evaluated. The estimate for the intercept indicates the estimate before training in the compound group (a and b), blocking group (c and d) or auto-blocking group (e and f).

Supplemental table S2. Information coded in the responses of DA neurons in the aversive learning model.

Stimulus	Before training	After training
US	1(US)	1 (US)
CS	0	0 [-1 (-USP)]*
CS+US	1 (US)	0 (USPE)

The table shows responses of DA neurons in the model shown in Fig. 4a to aversive US, CS and paired presentation of the CS and US before and after training. DA neurons govern enhancement of synaptic transmission that underlies conditioning (CS-CR synapse). After completion of training, paired presentation of CS and US does not produce responses in DA neurons and thus no further enhancement of synaptic transmission occurs. USP: US prediction; USPE: US prediction error. Responses are indicated as all or none (1 or 0). *Negative value in parentheses indicates inhibitory synaptic input.

Supplementary Figure S1

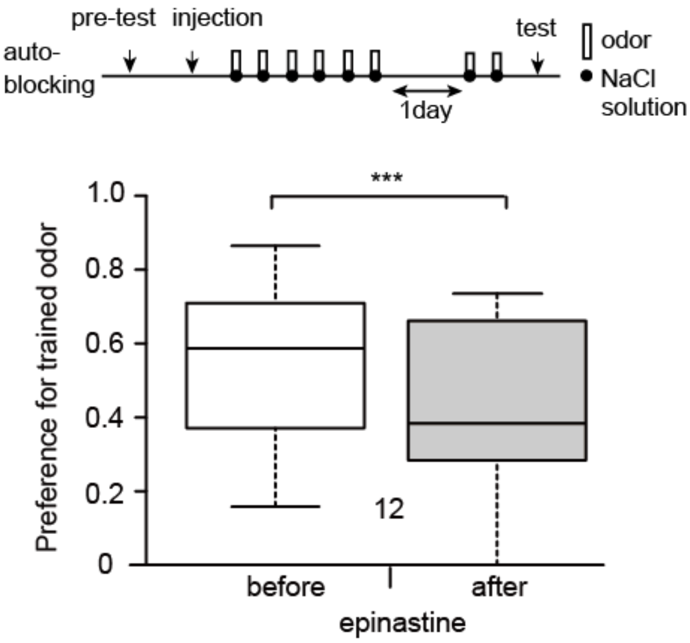

Figure S1. Auto-blocking does not occur by epinastine. One group of animals received a pre-test and was then injected with 3 µl of saline containing 2 µM epinastine. Thirty min later, they were subjected to 6-trial pairing of an odor with NaCl solution. The ITI was 5 min for. On the next day, they were subjected to 2-trial pairing of the odor with NaCl solution and 20 min later they received a post-test. Relative odor preferences for the trained odor before (while boxes) and after (gray boxes) training are shown as box and whisker diagrams. The number of animals is shown below the boxes. A GLMM was used for comparison of relative preferences for the trained odor before and after conditioning (Supplemental table S1). Statistical significance is shown as asterisks (*** $p<0.001$).

Supplementary Figure S2

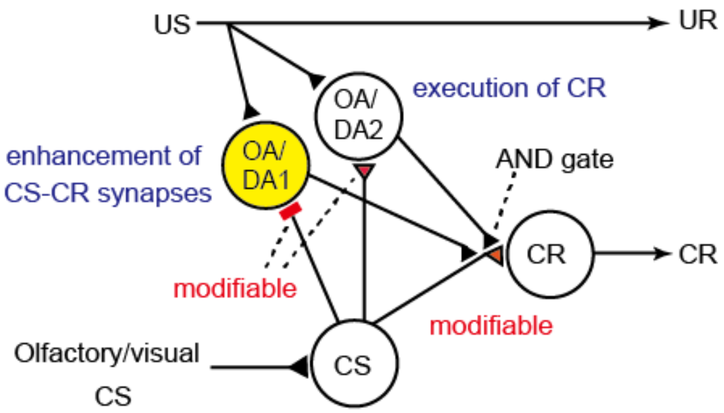

Figure S2. Complete description of our model of appetitive and aversive learning in crickets. The model was proposed to account for blocking and auto-blocking of appetitive learning[11] and aversive learning (this study), by modifying our previous model[15] that we proposed to accounts for our findings that blockade of OA- or DA-ergic transmission impairs learning and execution of conditioned response (or memory retrieval) in appetitive or aversive learning, respectively, in crickets[12-19]. The model assumes two classes of OA and DA neurons, namely, the OA1 and DA1 neurons ("OA/DA1" neurons; colored in yellow) that govern enhancement of "CS-CR" synapses (but not execution of CR) and "OA/DA2" neurons that govern execution of CR or memory retrieval (but not enhancement of "CS-CR" synapses). The model also assumes that (1) "CS" neurons (which may represent intrinsic neurons of the mushroom body) that convey signals about CS make silent or weak synaptic connections with dendrites of "CR" neurons (which may represent efferent (output) neurons of the mushroom body lobe), activation of which leads to a conditioned response (CR), but these synaptic connections are silent or very weak before conditioning, (2) The "OA/DA1" neurons are assumed to receive excitatory synapses that represent US signal and silent or very weak inhibitory synapses from "CS" neurons before training, which are strengthened by CS-US pairing. (3) During training, "OA/DA1" neurons receive excitatory synaptic input that represents actual US and inhibitory input from "CS" neurons that represents US prediction by CS, and thus their activities represent US prediction error signals (see legends of Fig. 4). (4) The "OA/DA2" neurons are assumed to receive excitatory synapses that represent US signal and silent or very weak excitatory synapses from "CS" neurons before training, which are strengthened by CS-US pairing. (5) "OA/DA2" neurons make synaptic connections with axon terminals of "CS" neurons, and coincident activation of "CS" neurons and "OA/DA2" neurons is needed for activation of "CR" neurons (AND gate) and for production of conditioned response. Presentation of CS after CS-US pairing activates "CS" neurons and then "OA/DA2" neurons and thus activates "CR" neurons to lead to conditioned response. UR: unconditioned response.
